# Obituary for a diagnosis: B‐cell prolymphocytic leukaemia (1974–2022)

**DOI:** 10.1002/hem3.35

**Published:** 2024-02-01

**Authors:** Stephen P. Hibbs, Matthew L. Smith, Deborah Swinglehurst

**Affiliations:** ^1^ Wolfson Institute of Population Health Queen Mary University of London London UK; ^2^ Department of Clinical Haematology Royal London Hospital London UK

Born in 1974 at the Royal Marsden Hospital in London,^1^ B‐cell prolymphocytic leukaemia was often referred to as ‘B‐PLL’ (*bee‐pul*) throughout their illustrious five‐decade career. During the 1970s and early 1980s, B‐PLL worked exclusively with their original supervisors, but later began collaborating with researchers in Italy,^2^ the Netherlands^3^ and Japan.^4^ In 1997, B‐PLL joined other prestigious diagnostic entities in the official canon of haematopathology: the World Health Organization's Classification of Haematological Malignancies,^5^ and proudly maintained their position through to the revised 4th edition of 2016.^6^

At the peak of their career, B‐PLL collaborated on hundreds of research projects, provided structure for the treatment of thousands of patients across the world, and appeared regularly in postgraduate haematology exams. Trainee haematologists were always advised to look out for B‐PLL who was clearly recognisable by their striking appearance but usually arrived unannounced. Depictions in medical textbooks inevitably focused on the large nucleolus that accompanied B‐PLL and that seemed to stare back from the slide when viewed under the microscope (Figure 1). While B‐PLL forged cordial, productive and durable alliances with researchers and clinicians, relationships with patients were challenging. Patients who encountered B‐PLL often experienced disabling symptoms and witnessed B‐PLL's stubborn refusal to cooperate with chemotherapy.

**Figure 1:**
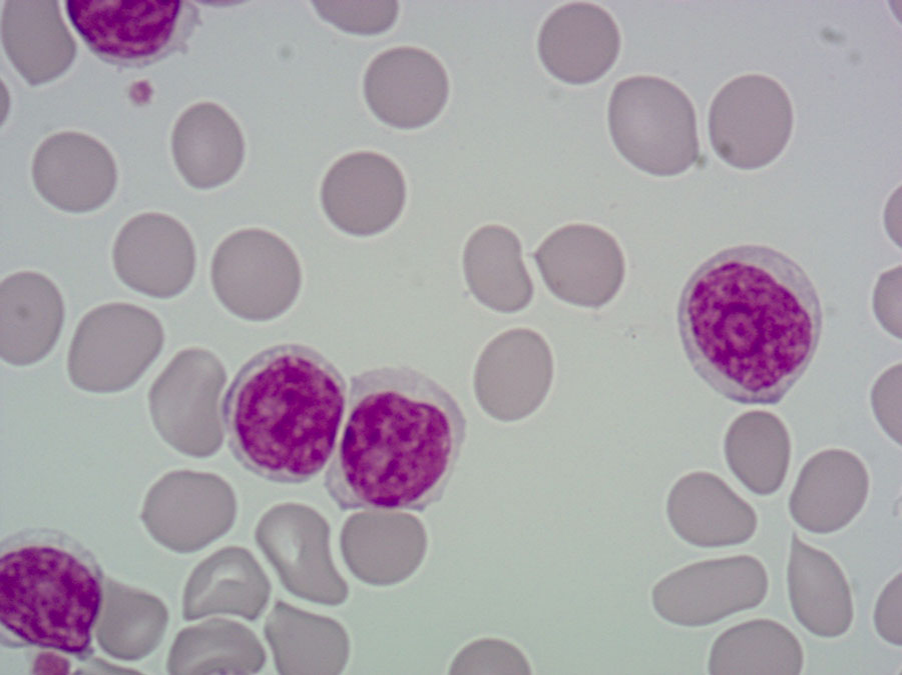
The famous ‘staring eyes’ of B‐cell prolymphocytic leukaemia, observable down the microscope (photograph taken by Dr. Matthew Smith).

Reports of concern for B‐PLL's health emerged in 2021, even as they continued to collaborate with scientists in France^7^ and Florida.^8^ Colleagues in haematopathology began to lose confidence in their ability to distinguish B‐PLL from other diagnostic entities, even with advanced genetic methods.^9^ In 2022, experts responsible for the World Health Organization classification system formally certified the demise of B‐PLL. Their departure coincided with that of the hairy cell leukaemia variant, a close associate. The clinical commitments previously fulfilled by B‐PLL have been transferred to one of their ex‐colleagues (mantle cell lymphoma) and two newly recruited entities with unusually long names.^10^

B‐PLL is survived by several diagnostic siblings, numerous patients who still carry its label and a medical worldview that stubbornly assumes the stability of diagnostic categories despite a convincing body of evidence that refutes this.^11^ Diagnostic labels are not timeless scientific truths, but instead reflect the understanding of a few people at a particular historical moment; they often have shorter lifespans than their human inventors.

## AUTHOR CONTRIBUTIONS

Stephen P. Hibbs conceptualized the article, wrote the initial draft and is the guarantor of the article. Matthew L. Smith and Deborah Swinglehurst critically reviewed the article and revised it. All authors agreed to the final version.

## CONFLICT OF INTEREST STATEMENT

All authors declare no conflict of interest.

## FUNDING

Stephen P. Hibbs is supported by a HARP doctoral research fellowship, funded by the Wellcome Trust (Grant number 223500/Z/21/Z).

## Data Availability

Data sharing is not applicable to this article as no data sets were generated or analysed during the current study.
